# Sleep Health Assessment and Treatment in Children and Adolescents with Chronic Pain: State of the Art and Future Directions

**DOI:** 10.3390/jcm11061491

**Published:** 2022-03-09

**Authors:** Emily F. Law, Agnes Kim, Kelly Ickmans, Tonya M. Palermo

**Affiliations:** 1 Department of Anesthesiology & Pain Medicine, University of Washington School of Medicine, Seattle, WA 98195, USA; tonya.palermo@seattlechildrens.org; 2 Center for Child Health, Behavior & Development, Seattle Children’s Research Institute, Seattle, WA 98121, USA; agnkim@augusta.edu; 3 Medical College of Georgia, Augusta University & University of Georgia Medical Partnership Campus, Augusta, GA 30912, USA; 4 Pain in Motion Research Group (PAIN), Department of Physiotherapy, Human Physiology and Anatomy, Faculty of Physical Education & Physiotherapy, Vrije Universiteit Brussel, Laarbeeklaan 103, 1090 Brussels, Belgium; kelly.ickmans@vub.be; 5 Movement & Nutrition for Health & Performance Research Group (MOVE), Department of Movement and Sport Sciences, Faculty of Physical Education and Physiotherapy, Vrije Universiteit Brussel, Pleinlaan 2, 1050 Brussels, Belgium; 6 Department of Physical Medicine and Physiotherapy, Universitair Ziekenhuis Brussel, Laarbeeklaan 101, 1090 Brussels, Belgium

**Keywords:** child, adolescent, pediatric, chronic pain, sleep, insomnia

## Abstract

Sleep is interrelated with the experience of chronic pain and represents a modifiable lifestyle factor that may play an important role in the treatment of children and adolescents with chronic pain. This is a topical review of assessment and treatment approaches to promote sleep health in children and adolescents with chronic pain, which summarizes: relevant and recent systematic reviews, meta-analyses, and methodologically sound prospective studies and clinical trials. Recommendations are provided for best practices in the clinical assessment and treatment of sleep health in youth with chronic pain. This overview can also provide researchers with foundational knowledge to build upon the best evidence for future prospective studies, assessment and intervention development, and novel clinical trials.

## 1. Introduction

Chronic pain is a major public health concern in children and adolescents, affecting up to 40% of youth [1,2]. Chronic pain in childhood can be nociplastic (i.e., arising from altered nociception in the central nervous system, such as fibromyalgia or central sensitization), as well as disease-related (i.e., arthritis, sickle cell disease) [1,2]. Headache, abdominal pain, and musculoskeletal pain are among the most common pain conditions in youth [1,2]. Across conditions, chronic pain in childhood is associated with decrements in children’s physical, social, and psychological functioning (i.e., increased anxiety and depressive symptoms), and low health-related quality of life [3,4,5,6,7,8,9,10]. Longitudinal studies demonstrate that having childhood chronic pain increases the risk for continuing chronic pain in adulthood, as well as limitations in educational and vocational attainment in adulthood [11,12,13,14,15,16]. Therefore, identifying factors that predict the development and maintenance of chronic pain in children is an urgent priority.

Physical health conditions and lifestyle factors (e.g., sleep, physical activity, obesity) are one set of vulnerability factors identified as important in chronic pain development and persistence [17]. In particular, sleep disorders and poor quality of sleep commonly co-occur with chronic pain in youth. In a systematic review, Valrie and colleagues [18] found strong evidence for increased sleep problems across samples of youth with both nociplastic and diseases-related chronic pain conditions in comparison with healthy controls. For example, over 50% of adolescents with chronic pain vs. 10% same-age adolescents without pain endorse having significant insomnia symptoms [19]. Further, children with chronic pain report poor sleep quality, have higher sleep anxiety, more bedtime resistance, more frequent awakenings during the night, and more daytime fatigue than the controls [18]. Many cross-sectional studies in adolescents and adults with a variety of chronic pain conditions indicate that sleep disturbances are associated with greater pain sensitivity, as well as greater disability, poorer quality of life, and greater healthcare use and costs [19,20,21,22]. There is not only substantial evidence that chronic pain can disrupt sleep, but also that sleep disturbances contribute to pain—a bidirectional association between sleep and pain has been described. 

**Figure 1:**
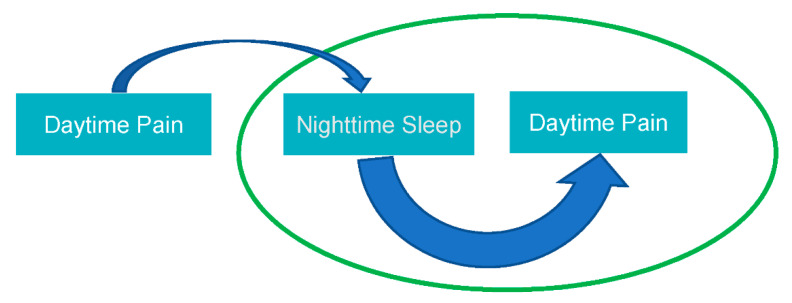
Direction of sleep–pain effects.

Finan and colleagues [23] synthesized the evidence for the bidirectional effects of sleep and pain in a systematic review. In the included studies that assessed unidirectional effects of sleep on subsequent pain, there was a general consensus that sleep disturbance could: (1) increase the risk for new incidences of chronic pain in pain free individuals, (2) worsen long-term prognosis of existing headache and chronic musculoskeletal pain, and (3) influence daily fluctuations in clinical pain. There was also complementing evidence that good sleep improves long-term prognosis of individuals with tension-type headaches, migraine, and chronic musculoskeletal pain. In the studies that assessed bidirectional effects of sleep and pain, findings suggested that the direction of sleep to chronic pain were more strongly supported than vice versa. For example, in one of the included studies by Lewandowski and colleagues [24], adolescents with disrupted sleep on a given day had increased pain on the subsequent day, yet the reverse direction of this relationship was not significant. Overall, the direction of sleep disturbances influencing subsequent pain was more consistently supported such as in Figure 1.

In this topical review, we use the term “sleep health” intentionally to highlight that sleep occurs along a continuum and is an important broad contributor to health and well-being. Sleep health is defined as “a multidimensional pattern of sleep-wakefulness, adapted to individual, social, and environmental demands, that promotes physical and mental well-being. Good sleep health is characterized by subjective satisfaction, appropriate timing, adequate duration, high efficiency, and sustained alertness during waking hours” [25].

This state of the art review aims to provide an overview of the role of sleep health in pediatric chronic pain, to present recommendations for clinical practice, and to provide a research agenda for designing future trials and prospective studies. Because studies consistently demonstrate that sleep disturbances are related to poor outcomes in children with chronic pain, including high pain-related disability and low health-related quality of life, sleep health has been proposed as a possible modifiable factor that may improve pain management for youth. We conducted a topical review to summarize the evidence for sleep health interventions in youth with chronic pain and also to highlight evidence in adult chronic pain, where gaps exist in the pediatric literature based on relevant and recent systematic reviews, meta-analyses, and methodologically sound prospective studies and clinical trials. We discuss future research needed to test the modulation of sleep as a potential therapeutic strategy for pain relief and prevention in children and adolescents.

## 2. Approaches for Sleep Health Assessment in Pediatric Pain Populations

Sleep health is a multi-dimensional construct, therefore a variety of approaches can be used in sleep assessment, including clinical interview, objective assessments, and self-report measures. In general, multi-method assessments of sleep health are recommended where possible in order to understand sleep patterns, sleep behaviors, perceptions of sleep quality, and experiences of daytime sleepiness. In a systematic review, de la Vega & Miro [26] identified three assessment procedures that have been used to assess sleep health specifically in adolescents with chronic pain: polysomnography, actigraphy, and questionnaires. 

Polysomnography (PSG) is an objective measurement tool, and should be considered when evaluation of sleep stages and/or sleep-related breathing is indicated [26]. Polysomnography is the gold standard for diagnostic assessment of physiological causes of sleep disturbance (e.g., obstructive sleep apnea, restless leg syndrome). PSG is usually conducted in a specialized hospital sleep laboratory and is typically limited to a single overnight assessment. There are some limited data on PSG to assess sleep in youth with [27] chronic pain, mostly in small samples with rheumatological conditions [28,29], and it is not yet clear how PSG may help with the evaluation or management of sleep complaints in youth with chronic pain. 

Daily sleep patterns in the adolescent’s home environment can be assessed using actigraphy, which is a watch-like device worn on the wrist to record motor movements using a continuous actimetry sensor [30,31,32]. Actigraphy has been used to describe habitual sleep–wake patterns (e.g., sleep duration, sleep efficiency, time awake after sleep onset) and to identify associations with other pain outcomes in youth with chronic pain [24,33,34,35]. In other pediatric populations, actigraphy has been used to identify sleep disturbances such as insomnia and hypersomnolence [32]. The most commonly used devices across studies include the Actiwatch 2 (Philips Respironics, Murrysville, PA, USA) and the MicroMini-Motionlogger (Ambulatory Monitoring, Inc., Ardsley, NY, USA). Unlike PSG, actigraphy does not evaluate sleep stages or sleep-related breathing. Limitations of actigraphy include possible misclassification of sleep–wake periods, where periods of high activity during sleep are erroneously classified as time awake and periods of low activity during wakefulness are erroneously classified as sleep. To address this challenge, a daily sleep diary should be used to validate periods of wake versus sleep on actigraphy [27,32,36]. There can also be variability between different devices, placements, and scoring algorithms [27,36,37]. A multi-modal assessment of sleep (e.g., combining actigraphy with self-report questionnaires) can also help to address these limitations. Another potential limitation is the cost to purchase and score actigraphy devices. While there are lower costs such as commercially available wearable devices (e.g., Fitbit), the reliability and the validity for the measurement of sleep patterns in pediatric populations are unknown. 

Self-report measures are useful for identifying behavioral factors that are contributing to sleep disturbances and can be used alone or in combination with objective measurements. Three self-report questionnaire measures have been identified as “well-established” for children and adolescents with chronic pain [26]: the Adolescent Sleep Wake Scale (ASWS) [38], the Adolescent Sleep Hygiene Scale (ASHS) [38], and the Children’s Sleep Habits Questionnaire (CSHQ) [39] (see Table 1). The ASWS and the ASHS are adolescent self-report questionnaires that assess more targeted areas of behavioral sleep disturbances, including perceived sleep quality (ASWS) and sleep habits (ASHS). One limitation of these tools is the number of items and the length of administration, which are burdensome. To address this barrier, Essner and colleagues [40] proposed a 10-item short-form version of the ASWS (ASWS-SF) based upon exploratory factor analyses with a broad sample of youth with chronic health conditions, including youth with chronic pain, which has demonstrated adequate reliability and validity in subsequent studies of youth with chronic pain and co-occurring sleep disturbances [41,42]. The CSHQ is a multidimensional parent-report measure that can be useful for screening for a wide range of medical and behavioral sleep disorders in younger children.

Historically, a major gap in sleep assessment for youth has been the lack of brief developmentally informed self-report tools to screen for insomnia symptoms. Insomnia is characterized by persistent dissatisfaction with sleep quantity or quality that is associated with difficulty falling asleep, maintaining sleep, and/or early morning waking which results in daytime impairment [43]. Screening for insomnia is particularly important for youth with chronic pain, given the high prevalence of insomnia symptoms in this population [19]. To address this gap in insomnia measurement, Bromberg et al., [44] developed the 13-item Adolescent Insomnia Questionnaire (AIQ) and demonstrated acceptable psychometric properties in a heterogeneous sample of adolescents with chronic pain and other chronic health conditions. The ASWS-SF and AIQ are well-suited for use in clinical and research settings due to their brevity, ease of scoring, and established reliability and validity in pediatric chronic pain populations (see Table 1).

**Table 1:** Recommended self-report questionnaire assessments of sleep health for children and adolescents with chronic pain.

Measure Name	Domain	Age Range	Reporter	Items/Subscales	Primary Citation
Adolescent Sleep Wake Scale (ASWS)	Sleep quality	12–18 years	Youth	28 items, yields a total score and 5 subscale scores (Going to Bed, FallingAsleep, Maintaining Sleep, Reinitiating Sleep, Returning to Wakefulness)	LeBourgeois et al., [38]
Adolescent Sleep Wake Scale Short Form (ASWS-SF)	Sleep quality	12–18 years	Youth	10 items, yields a total score and 3 subscale scores (Falling Asleep and Reinitiating Sleep, Returning to Wakefulness, Going to Bed)	Essner et al., [40]
Adolescent Sleep Hygiene Scale (ASHS)	Sleep habits	12–18 years	Youth	28 items, yields a total score and 9 subscale scores (Physiological, Cognitive, Emotional, Sleep Environment, Daytime Sleep, Substances, Bedtime Routine, Sleep Stability, Bed/Bedroom Sharing)	LeBourgeois et al., [38]
Children’s Sleep Habits Questionnaire (CSHQ)	Sleep disorders screen	4–10 years	Parent	45 items, yields a total score and 8 subscale scores (Bedtime Resistance, Parasomnia, Sleep Onset Delay, Sleep Duration, Sleep Anxiety, Night Wakings, Sleep-Disordered Breathing, Daytime Sleepiness)	Owens et al., [39]
Adolescent Insomnia Questionnaire (AIQ)	Insomnia screen	11–18 years	Youth	13 items, yields a total score and 3 subscale scores (Sleep Onset, Sleep Maintenance, Sleep Dissatisfaction and Impairments)	Bromberg et al., [44]

## 3. Interventions to Improve Sleep Health in Pediatric Pain Populations: Cognitive-Behavioral Therapy for Pain Management, Cognitive-Behavioral Therapy for Insomnia, and Sleep Hygiene Education

In general, psychological and behavioral treatments have been used to improve sleep health in children and adolescents [45,46,47]. There is limited rationale or evidence to use pharmacotherapy to address sleep health in youth, especially when the most common concerns center around behavioral insomnias [48,49]. 

Cognitive-behavioral therapy for pain management (CBT-Pain) is the gold standard psychological intervention for youth with chronic pain [50,51]. CBT-Pain incorporates training in cognitive skills, relaxation and distraction methods, and parent operant strategies in order to support adaptive coping with pain and participation in normal daily activities [51]. Although sleep health is recognized as an important outcome for pediatric chronic pain treatment [52], sleep has been rarely assessed or specifically targeted in psychosocial interventions. When sleep intervention is included, it is typically brief (one session or less) and focused on sleep hygiene education [53]. Sleep assessment in trials of CBT-Pain has also been limited, typically to a single modality (i.e., self-reported sleep quality or actigraphy). 

A recent systematic review by Klausen et al., [54] found that sleep was reported as a treatment outcome in only two published RCTs of CBT-Pain in pediatric samples [55,56]. Sleep assessments included a self-report measure of sleep quality [56] and seven days of actigraphy monitoring [55]. Results were mixed. Findings from one trial indicated a small but significant benefit from CBT-Pain on sleep quality relative to pain education control [56], while the second trial found no difference in sleep duration or sleep efficiency on actigraphy between CBT-Pain and usual care [55]. The different pattern of findings between these two trials may reflect methodological differences in sleep assessment, as each was limited to a single assessment modality (i.e., self-reported sleep quality [56] and actigraphy [55]). In both trials, intervention specifically targeting sleep was limited to a brief education about sleep hygiene.

Cognitive-behavioral therapy for insomnia (CBT-I) is recommended by the American Academy of Sleep Medicine as a first line treatment for adults with sleep disturbances [57]. Core treatment strategies include education about sleep and sleep hygiene, stimulus control, and sleep restriction [57]. The overarching goal of treatment is to develop a consistent sleep–wake schedule and strengthen the association between bed and sleep by limiting time awake in bed. A recent systematic review identified 10 RCTs of CBT-I for adults with chronic pain and comorbid insomnia, some of which only delivered CBT-I while others delivered CBT-I followed by CBT-Pain [58]. Results of meta-analyses demonstrated large positive benefits of CBT-I on global measures of sleep at post-treatment (SMD = 0.89) and follow-up (SMD = 0.56) [58]. Small improvements in pain at post-treatment (SMD = 0.20) were also identified, but this was not maintained at follow-up [58]. Importantly, many of these prior trials have significant limitations, including small sample sizes, short follow-up periods and inadequate assessment of pain outcomes. Findings from two large rigorous trials with older adults with insomnia and comorbid osteoarthritis pain suggest that improvements in sleep in response to CBT-I may lead to both short- and long-term improvements in pain [59,60]. However, neither trial was designed to empirically test temporal associations between the sleep–pain relationship and more research is needed. 

We are not aware of any published RCTs that evaluated the safety or efficacy of CBT-I in youth with chronic pain conditions. There is one published RCT which compared the efficacy of sleep hygiene guidelines to usual care in adolescents with migraine and co-occurring poor sleep health [61]. Results indicated that compared with usual care control youth who received the sleep hygiene guidelines had a greater reduction in migraine frequency from pre-treatment to 3-month and 6-month follow-ups [61]. However, this study was limited by the relatively small sample size (total n = 70) and lack of validated assessment tools measuring headache and sleep outcomes. 

While controlled trials evaluating efficacy of CBT-I in pediatric pain populations are lacking, there are a few single arm pilot trials in mixed samples of adolescents with chronic pain and other medical and mental health comorbidities [41,62]. These studies used brief 4–6 session CBT-I protocols delivered to youth and their parents following standard core treatment elements, including education about sleep and sleep hygiene, stimulus control, and sleep restriction. Results of this pilot work demonstrated feasibility for in-person treatment delivery and preliminary efficacy, where youth showed improvements in sleep quality, sleep hygiene, sleep duration, sleep efficiency, and mood and anxiety symptoms [41,62].

Hybrid CBT programs have also been developed for adults and youth with co-occurring chronic pain and insomnia, which combine CBT-I with CBT-Pain. By providing treatment for two problems simultaneously, hybrid CBT may offer some practical advantages for both patients and healthcare systems. A recent topical review found emerging evidence for the feasibility, acceptability, and preliminary efficacy of hybrid CBT programs for improving pain and sleep outcomes in adults with coexisting chronic pain and insomnia [63]. However, these studies are limited to a handful of relatively small single-arm pilot trials [64,65,66]. We are not aware of any published randomized controlled trials evaluating hybrid CBT protocols for youth with comorbid insomnia and chronic pain, although related work is underway in adult chronic pain populations (e.g., [67]).

To address this gap, Law and colleagues [42] developed a six-session hybrid CBT program delivered in person to youth with chronic migraine and comorbid insomnia symptoms (see Table 2). The treatment protocol integrates core components of CBT-I (sleep restriction, stimulus control, sleep hygiene) with core components of CBT-Pain (relaxation and distraction methods, cognitive skills, and parent operant training). In a single-arm pilot trial, preliminary evidence for efficacy was demonstrated on improvements in sleep quality, sleep hygiene, sleep duration, and sleep efficiency. Notably, youth also experienced reductions in headache-related disability and headache frequency from pre- to post-hybrid CBT treatment [42].

Given the lack of behavioral sleep medicine specialists in many communities, there is also interest in digital health interventions (e.g., mobile apps, internet interventions) to improve sleep health. There is robust evidence indicating that self-guided web-based CBT-I produces similar effect sizes for improving insomnia symptoms in adults compared to face-to-face CBT-I [68], although similar data in youth are lacking. Recently, Carmona and colleagues [69] demonstrated feasibility and acceptability of a transdiagnostic web-based app for adolescents and young adults (AYAs), which provides self-guided training in sleep education, personalized feedback comparing the user’s sleep patterns to age-based norms, and tailored goal setting to improve sleep habits. We are not aware of any published randomized controlled trials testing technology delivered sleep health interventions for youth, although studies are currently underway (e.g., [70]). 

## 4. Future Directions for Clinical Practice

Our review uncovers a number of areas important to consider in clinical practice (see Table 3). Given evidence of the high prevalence of sleep disturbances among youth with chronic pain and the importance of overall sleep health on pain and well-being, routine screening should be implemented. In addition to the clinical interview, there are brief validated self-report screening measures to assess insomnia, sleep quality, and sleep impairment in youth, which should be used. The assessment of sleep health in all children and adolescents presenting with chronic pain is recommended. Interventions targeting sleep hygiene and insomnia symptoms can be offered to youth; this may include education about sleep needs, importance of consistency in sleep–wake schedules, and tips for healthy sleep (e.g., establish a positive bedtime and waking routine, limit electronics in the bedroom). Additional consideration may be needed to tailor education to the unique challenges that impact sleep health in youth with chronic pain such as a lack of scheduled activities and routines, low levels of physical activity, and the use of napping as a coping strategy for pain management. Youth with clinically significant insomnia symptoms should be referred to a sleep specialist. Once the efficacy and the safety of insomnia interventions are established in youth with chronic pain, considerations for implementation would include the use of telehealth and digital health technologies to improve access and potentially reduce costs. 

## 5. Future Directions for Research

Our review also highlights the need for research in multiple areas of sleep health in adolescents with chronic pain (see Table 4). First, there is an incomplete understanding of the impact of pain treatments on sleep outcomes in youth with chronic pain as sleep is not often measured in pain clinical trials. Knowledge of the safety and efficacy of sleep treatments such as CBT-I and Hybrid CBT-I/CBT Pain has not yet been fully established in youth with chronic pain. In particular, future research of sleep treatments using randomized controlled trial designs with long-term follow-up is needed. Sleep has been shown to influence subsequent pain, therefore research to understand the optimal sequence of pain and sleep interventions is needed. In particular, it will be important to evaluate whether intervening to improve sleep first may boost the effects of subsequent pain interventions. There are also gaps in the understanding of the longitudinal and causal relationships between sleep health and chronic pain, including whether there are key vulnerability periods (e.g., puberty) that may influence the linkage between sleep and pain. Few studies have focused on how positive aspects of sleep health may influence pain and pain management in youth. Furthermore, there is limited understanding of how sleep health influences motivation and self-efficacy among youth with chronic pain and how this may influence their ability to engage in pain self-management behaviors. Another future direction for research is to understand sociodemographic influences on sleep health, including possible disparities in the impact of sleep health on youth. Last, it will be important to identify shared biopsychosocial mechanisms that underlie the treatment benefits of pain and sleep interventions for youth to better inform optimization of these interventions in the context of comorbid pain and sleep problems.

## 6. Conclusions

There is growing consensus among experts that sleep health has a direct effect on pain perception, pain intensity, and pain-related disability among youth with chronic pain. Validated assessment tools that can be considered in clinical practice and research settings vary in terms of their cost and burden, and include polysomnography, actigraphy, and self-report questionnaire measures. Appropriate assessment tools have been developed and validated for pediatric populations with chronic pain, which can be used in clinical practice and research studies. Sleep health can be modified through psychological and behavioral interventions. Although randomized controlled trials of psychological interventions specifically targeting sleep health in youth with chronic pain are limited, a growing number of pilot studies supports the feasibility and preliminary efficacy of sleep hygiene education and cognitive-behavioral therapy for insomnia (CBT-I) for improving sleep patterns, improving perceived sleep quality, and reducing pain and pain-related disability in adolescents and young adults with chronic pain when delivered face-to-face and via digital health technologies. Clinicians are encouraged to routinely screen sleep health in all children and youth presenting with chronic pain. Research is still needed to characterize the sleep–pain relationship over time in youth with chronic pain to identify mechanisms that account for their interrelationship and to definitively evaluate the safety and the efficacy of psychological interventions targeting sleep health in pediatric chronic pain populations.

## Figures and Tables

**Table 2:** Best evidence psychological interventions for addressing sleep health in youth with chronic pain.

Intervention	Target Population	Level of Evidence	Setting of Care Delivery	Provider Discipline
Sleep hygiene education	Youth with chronic pain	Promising	Tertiary care clinic (e.g., Pain Medicine Clinic, Sleep Clinic), Digital health technology	Psychologist, Behavioral Sleep Specialist, Pain medicine specialist
Cognitive-Behavioral Therapy for Insomnia (CBT-I)	Youth with comorbid chronic pain, insomnia, and mental health conditions	Promising	Tertiary care clinic (e.g., Pain Medicine Clinic, Sleep Clinic), Self-guided digital health technology	Psychologist, Behavioral sleep specialist

**Table 3:** Future Directions for Clinical Practice.

1.	Integrate screening for sleep disturbances into the assessment of all children and adolescents presenting with chronic pain.
2.	Provide sleep health interventions to target sleep hygiene and insomnia in youth presenting with sleep disturbances.
3.	Where available, use technology to deliver sleep health treatments, e.g., via telehealth and digital health technologies.
4.	Disseminate evidence-based sleep interventions.
5.	Before considering approaches to dissemination and implementation, further work is needed to understand safety and efficacy of CBT-I and Hybrid CBT-I/CBT-Pain interventions for youth with chronic pain via controlled trials.

**Table 4:** Future Directions for Research.

1.	Comprehensively characterize the impact of pain treatments on sleep health in youth with chronic pain.
2.	Evaluate the safety and efficacy of CBT-I and Hybrid CBT-I/CBT-Pain interventions for youth with chronic pain and co-occurring sleep disturbances.
3.	Conduct research to understand optimal sequencing of pain and sleep interventions, in particular to understand whether children and adolescents may benefit synergistically from improvements in sleep prior to beginning pain self-management interventions.
4.	Conduct longitudinal studies to identify the causal relationship between sleep health and chronic pain over time to uncover mechanisms and identify key vulnerability periods.
5.	Characterize resiliency in sleep health and how this can be enhanced among youth with chronic pain.
6.	Understand sociodemographic influences on sleep health among youth with chronic pain.
7.	Understand how sleep health influences motivation and self-efficacy among youth with chronic pain.
8.	Identify shared biopsychosocial mechanisms that underlie treatment benefits of pain and sleep interventions for individuals with co-occurring conditions.

## Data Availability

Not applicable.
